# Case Report: Secukinumab for the treatment of severe psoriasis in a patient with hereditary hemochromatosis

**DOI:** 10.3389/fmed.2025.1594640

**Published:** 2025-08-08

**Authors:** Yingzhe Yu, Lingyi Lu, Xin Fan, Sihan Wang, Bingjiang Lin

**Affiliations:** The First Affiliated Hospital of Ningbo University, Ningbo, China

**Keywords:** psoriasis, hereditary hemochromatosis, secukinumab, liver function, IL-17A

## Abstract

There are several comorbidities associated with psoriasis, including genetic disorders such as hereditary hemochromatosis, which can lead to organ damage secondary to iron overload. Herein, we report the case of a 38-year-old Chinese man with hereditary hemochromatosis who received secukinumab for the treatment of severe psoriasis. Follow-up after 3 months showed that the patient’s lesions had almost resolved and remained well-controlled for 2 years without any reported side effects. Patients with psoriasis and hereditary hemochromatosis have limited treatment options due to the effects of iron overload on the liver, particularly because it may increase the risk of hepatocellular carcinoma. Interleukin-17A (IL-17A) inhibitors, such as the secukinumab used in this case, may benefit these patients.

## Introduction

Psoriasis is a chronic and recurrent inflammatory skin disease, affecting more than 60 million people worldwide. It results from a combination of genetic susceptibility and environmental triggers (1). Studies have shown that psoriasis is associated with a variety of comorbid conditions, such as metabolic syndrome, cardiovascular disease, depression, inflammatory bowel disease, and chronic kidney disease (2). In addition, several reports have documented cases of psoriasis occurring in combination with genetic diseases, including hemochromatosis and Hermansky–Pudlak syndrome (3, 4).

## Case description

**Figure 1:**
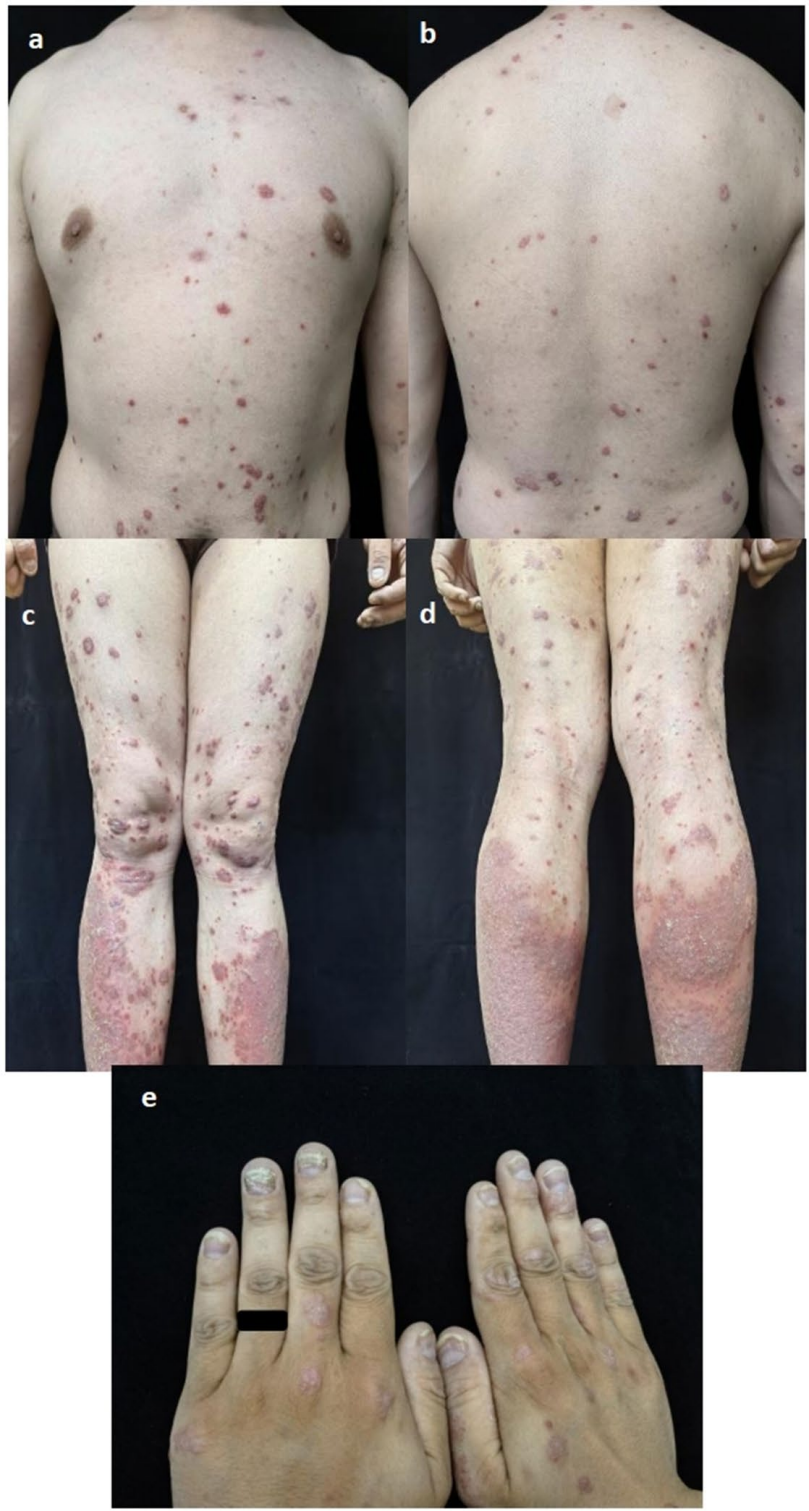
**(a–d)** Clinical manifestations prior to secukinumab on trunk and mbs with PASI score of 12.2; **(e)** nails presentation including pitting. Eukonychia, oil drop discoloration with NAPSI score of 28 before treatment.

**Figure 2:**
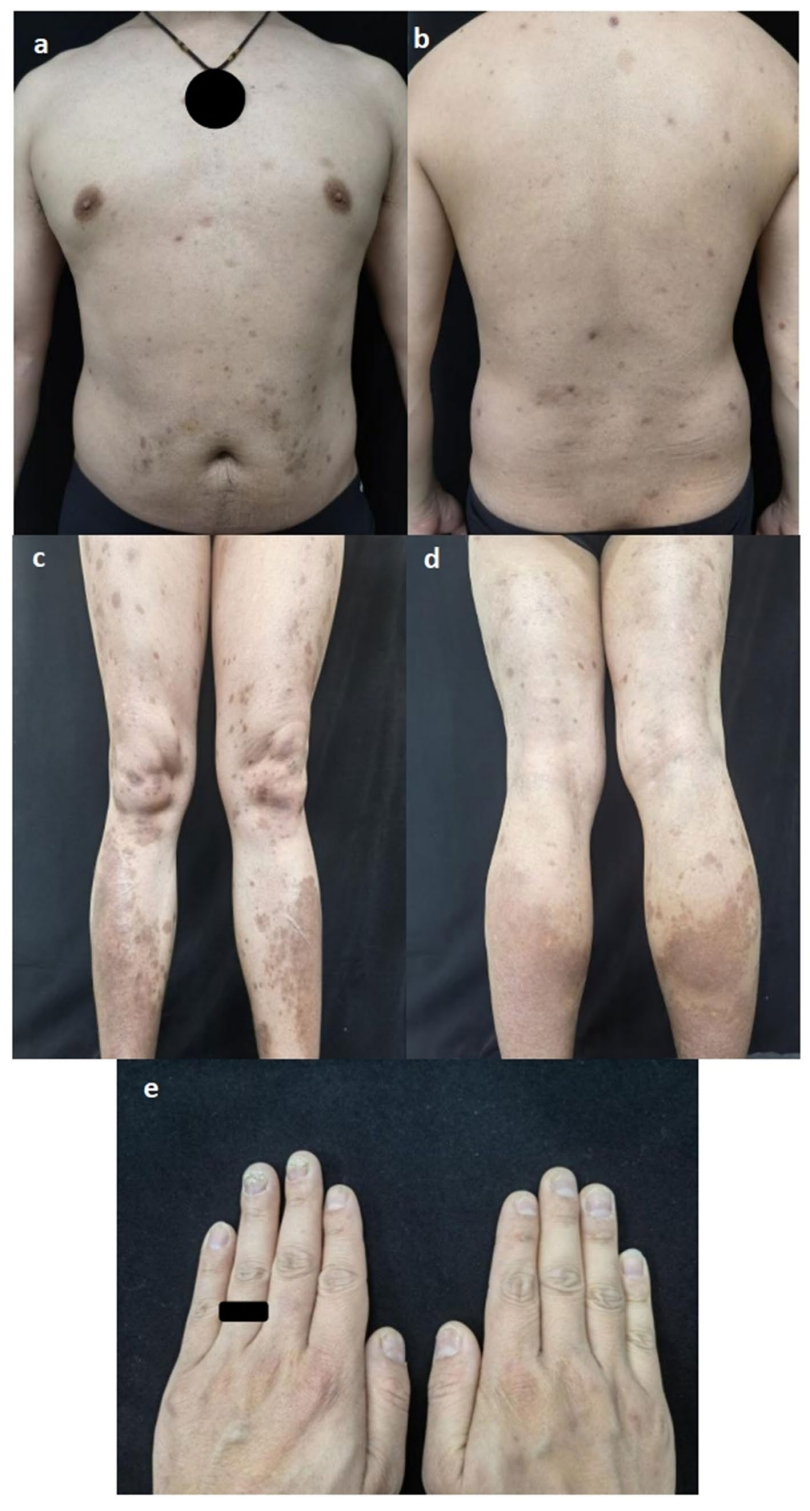
**(a–d)** The lesions of trunk and extremities were replaced by hyperpigmentation with PASI score of 0.4 after 3 months treatment; **(e)** pitting, leukonychia, oil drop discoloration of nails was improved with NAPSI score of 9 after treatment.

**Figure 3:**
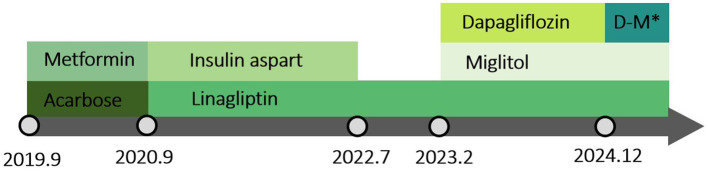
Timeline of the medications administered during secukinumab treatment. *D-M, Dapagliflozin-Metformin.

A 38-year-old Chinese man had suffered from severe psoriasis for 15 years and had been treated intermittently with ultraviolet light, topical glucocorticoids, topical calcipotriol, and herbal remedies. Two years ago, he was hospitalized due to malaise and twitching of the hands and feet that had persisted for 1 month. Shortly afterward, the patient was diagnosed with hereditary hemochromatosis, liver failure, hepatic cirrhosis, and diabetes mellitus based on the following findings: elevated aspartate aminotransferase (41 U/L; normal <40 U/L), fasting blood glucose (10.48 mmol/L, normal value <6.10 mmol/L), serum ferritin (16.19 μmol/L; normal value <11.98 μmol/L), and AFP (36.6 μg/L; normal value <20 μg/L); hepatobiliary MRI showing cirrhosis, splenomegaly, and portal hypertension with collateral circulation; and gene detection revealing missense mutations in *BMP2*: c.869A > C and c.393A > T. The proband’s parents and his only daughter were all asymptomatic. In addition, his daughter’s genetic testing showed no mutations. Serum ferritin and liver enzymes improved after symptomatic treatment, while cirrhosis and diabetes persisted. Meanwhile, psoriasis therapy was paused, resulting in worsening rashes with a Psoriasis Area and Severity Index (PASI) score of 12.2 (Figures 1a–d) and a Nail Psoriasis Severity Index (NAPSI) score of 28 (Figure 1e). As the lesions were distributed on exposed areas, directly affecting the patient’s quality of life, he reported a Dermatology Life Quality Index (DLQI) score of 23. With normal liver and renal function, normal infection markers, a slightly elevated AFP (32 μg/L; normal value <20 ng/mL), a negative nail fungus examination result, and evidence of liver cirrhosis on abdominal MRI, the patient was finally treated with secukinumab (300 mg every week for 4 weeks, followed by 300 mg every 4 week) under close follow-up. In addition, 3 months later, the lesions on the trunk and extremities had resolved, leaving only hyperpigmentation, with the PASI score decreasing from 12.2 to 0.4 (Figures 2a–d) and the DLQI score from 23 to 7. Simultaneously, pitting, leukonychia, and oil drop discoloration of the nails improved, with the NAPSI score decreasing from 28 to 9 (Figure 2e). No adverse effects were observed during follow-up visits at 2 weeks, 3 months, and up to 2 years. Antidiabetic therapy was maintained throughout (Figure 3).

## Discussion

Hereditary hemochromatosis, a genetic disorder characterized by dysregulation of iron homeostasis, has been extensively studied in European populations, while research on Asian populations remains limited (5). Hereditary hemochromatosis manifests with a range of clinical symptoms—including cirrhosis, hepatomegaly, hepatocellular carcinoma, skin pigmentation, impaired memory, chronic fatigue, weakness, arthritis, loss of body hair, diabetes, and heart failure—resulting from iron overload in multiple organ systems (6, 7).

Herein, we presented a rare case of a patient with coexisting severe psoriasis and hereditary hemochromatosis, with iron overload mainly affecting the liver. To the best of our knowledge, there is still a gap in studies on psoriasis comorbid with hereditary hemochromatosis in Asian populations. In particular, for patients presenting with liver injury, safety concerns exist regarding conventional therapy. For the current patient, a young adult male with hereditary hemochromatosis, there was a significant risk of cirrhosis and hepatocellular carcinoma, both of which can be life-threatening. Nowadays, secukinumab—an anti-IL17A biologic agent—is considered an effective and safe treatment for moderate-to-severe psoriasis. In this case, the patient showed improvement in both cutaneous and nail lesions following secukinumab treatment, although nail improvement progressed more slowly than skin lesion improvement during the 3-month follow-up observation, consistent with previous research (8). In addition, IL-17A not only plays an important role in psoriasis but is also associated with hepatic diseases such as hepatocellular carcinoma and non-alcoholic fatty liver disease. Therefore, secukinumab has been identified as a potential supplementary therapeutic option for hepatocellular carcinoma (9). IL-17A has been proven to be associated with the progression of hepatocellular carcinoma (10). Studies have shown that IL-17A promotes carcinogenesis by inhibiting autophagic cell death in hepatocellular carcinoma and stimulates the invasion-metastasis cascade by activating AKT signaling (9, 11).

To date, we have followed the patient for 2 years, during which no progression of cirrhosis or any evidence of hepatocellular carcinoma has been observed. However, more cases, longer follow-up periods, and further exploration of potential mechanisms are needed.

## Conclusion

The combination of severe psoriasis and hereditary hemochromatosis, which is rare in Asian populations, presents a therapeutic challenge due to multisystem involvement. In particular, for patients at risk of hepatocellular carcinoma, IL-17A inhibitors have proven to be an effective and safe treatment option.

## Data Availability

The datasets presented in this article are not readily available because data are available from the corresponding author upon reasonable request. Requests to access the datasets should be directed to BL, linbingj@163.com.
